# Bread and Pellagra

**Published:** 1916-08

**Authors:** J. R. Lowery

**Affiliations:** Raleigh, N. C.


### Bread and Pellagra.

BY J. R. LOWERY, M. D., RALEIGH, N. C.

Pellagra is a disease which, a dozen years ago, had never been heard of by 99 per cent of the physicians of this country. Today it is killing more people than any one disease in the South. What is the cause of it? Is it due to some infectious or contagious germ? Statistics and men of national reputation say no. Then it is due to some condition that exists now, that did not exist a number of years ago.

Some will tell you that it is due to an open privy. We have had an open privy in this country since Columbus landed on our virgin soil, but none of his followers ever heard of pellagra for about four hundred years.

Some will say that it is due to nitrogenous diet, but we are eating just as much nitrogenous food today as we ate half a century ago, and we did not have pellagra then. The only change we have made in our diet is the bread that we eat.

Half a century ago, the bread eaten by the people of the South was made from burr mill flour that contained much indigestible bran. They ate this bread three hundred and sixty-five days in the year, when they ate flour bread at all. Many of the poorer people had only bread made from corn meal which contained more indigestible substance than the flour bread. Today, these same people are eating bread from the flour made by the roller process, which contains practically no indigestible substance; therefore, leaves no residue.

As soon as we began to eat food that did not leave enough residue to keep the intestines properly distended, they became twisted upon themselves and formed kinks, which is the cause of the now famous Lane's kinks. I know of no better way of illustrating this condition than by taking an automobile tire. Put only a small amount of air in it, and it will easily kink; but when you fully distend it, it is impossible to put a kink in it. The kinking of the bowel produces stagnation and pocket formation. The bowels may move everyday, but still there will be fecal matter in the pockets of these kinks that will remain there for days.

Whenever we have stagnation for a number of days, in any part of the digestive tract where there is no free hydrochloric acid, then we have the growth and development of lactic acid forming bacilli. This is seen in cancer of the pyloric region of the stomach. The cancer destroys the acid forming glands of the stomach; at the same time producing stagnation by obstruction at the pylorus. When we have this condition, we always find lactic acid in the stomach.

In other rare conditions of achylia with thickening of the mucous membrane of the pyloric region of the stomach so as to cause stagnation, we also find lactic acid. This obstruction does not have to be complete, but only enough to keep the stomach from completely emptying itself. There remains a small amount of food in the stomach all the time, although 90 per cent of the food remnants may pass from the stomach daily.

Now, the acidoses that causes pellagra is not the lactic acid that is formed in the stomach under comparatively rare pathological conditions, but it can be easily seen that if lactic acid forms in the stomach under any condition where we have obstruction and no hydrochloric acid, that all forms of acid-forming bacteria will grow in the stagnated fecal matter of the intestines and produce an acidoses.

The fecal matter in the intestines may get into these kink pockets and remain for days and the bowels move every day. In these pockets, millions of bacteria are found which form acid all the time which is absorbed, carried through the body and produces a general acidoses, causing symptoms which we call pellagra by irritating various tissues of the body. These poisons come out through the sweat, and as soon as the rays of the sun come in contact with it the water of the sweat is evaporated, causing a concentration of the acid to such an extent that it makes the skin die and peel off; and if continued for any length of time, the hands and face will become sore. This condition looks like an acid burn; and if treated like a burn it will get well as far as the skin is concerned. The best local application that I have tried being equal parts of linseed oil and lime water. The lime water is an alkali and the oil acts as a protection to the skin.

Now, in regard to the Lane's kinks, heretofore spoken of, we all know that after an operation for Lane's kinks the patient is better for awhile; but, in most cases, the same old symptoms return in from six to twelve months. This is due to the patient not eating food that will leave enough residue to keep the bowels properly distended, and they kink again. If after the operation were put on a high residue leaving diet the bowels would be properly distended and the kinks would not return.

In a few generations the intestines will degenerate from non-use and become so small that the amount of residue that we now have will fill them up. Then pellagra and Lane's kinks will be things of the past.

Now, there are other forms of stagnation of a minor degree in which the fecal matter is not pocketed, and does not remain stagnant long enough for acid to form; but just long enough for an excessive amount of the products of alkaline bacteria to form and be absorbed. Under these conditions, lactic acid-forming bacilli given either in tablets or culture gives good results.

There are not enough poisons of any kind in the healthy human being to cause the pellagrous symptoms. It is only seen in people who have had their resistance lowered from such causes as: poor food, overwork, childbirth, anemia, la grippe, malaria and many other causes. For this reason, all people who eat bread made from white flour and have intestinal stasis do not have pellagra; and for this reason the poor people are more subject to it than are the rich. They have poorer food, and have to endure many more hardships than the rich.

Dr. Goldberger of the United States Health Service says that pellagra is due to the lack of nitrogenous diet, such as beef, eggs, milk and beans. Now, I would like for him to tell us what poison is formed by the lack of the above diet that is so severe as to cause hundreds of deaths, and many more cases of insanity. I would also like for him to tell us why it is that a great number of pellagra patients come from the farm, and are consuming the same amount of fresh eggs, milk and beans as our farmers have always consumed, while the symptoms of pellagra did not appear until roller flour was introduced into the South.

The feeding of great quantities of milk, eggs and beans may, and in my opinion will, cure some cases by making the patient stronger and raising the resisting power of the body, thereby enabling it to throw off these poisons, for, as I have already said, you never find this disease in a person who was perfectly healthy before the first appearance of the symptoms of pellagra.

The diet with which Dr. Goldberger cures pellagra is the same that we give to our tubercular patients, and many of them get apparently well. But who will claim that tuberculosis is due to the lack of milk, eggs and other nitrogenous foods?

Dr. Goldberger has produced pellagra in convicts by feeding them on a diet that contained no nitrogenous foods; but my opinion is that he could have perished them to death if he had kept their intestines flushed out with bran biscuits and castor oil, and they would not have developed the pellagrous symptoms.

I will admit that the diet is the most essential part of the treatment of pellagra, as it is in every disease; but we can cure many cases by giving them medicine to help out nature and the diet, that would otherwise die, for diet cannot accomplish everything. Of the many drugs that have been tried in the treatment of this dread disease, arsenic, given hypodermically, stands at the head of the list.

Hypodermics of arsenic do not cure the disease any more than the nitrogenous diet does. It merely stimulates the cells of the body, thereby raising the resisting power of the body and enabling the patients to resist these poisons.

Sometimes one will give too much arsenic and overstimulate the tissues and produce an irritation. I have given one dose of neosalvarsan and in ten days a second, then all the symptoms would disappear; but when I gave the third the skin and mucous membrane would become red and inflamed again. This was due to overstimulation by arsenic. Sometimes this redness would go all over the body and the skin would peel off, but in most cases the redness and irritation of the skin would only appear where the lesions of the pellagra were. When I gave enough to produce irritation, results were, invariably, good.

Now, if we wish to eliminate pellagra from the South, we must get back to nature's way of living. After a case is developed, we must raise the resistance of the body, either by giving strong and fattening diet, or by a more rapid and certain method of giving arsenic, either by the mouth, in the muscle or in the vein; at the same time putting them on whole wheat bread.

[Dr. Lowery, of Raleigh, N. C., contributes a most excellent paper in which he shows that bolted flour is at least under suspicion. Fletcherizers may chew their food in vain, if their bread is "bolted" they may have pellagra. The doctor gives as the cause of pellagra bread made from flour or meal without bran. He and the vitamin theory mentioned by Dr. Buck are apparently to have a collision or at least a collusion, but not so. Dr. Lowery says naught of vitamins, he wants the bran just for "roughness.". Why not have a little alfalfa? And how is it that the people of the North have eaten bread made from bolted flour so much longer than we of the South without pellagra? Dr. Lowery honors Dr. Goldberger by accepting the Jackson, Mississippi, experiment as successful in producing pellagra. I honestly believe that Dr. Goldberger honestly believes that, too. But I do not. The men under experiment had been working: working men chafe. All of the striking lesions were on their scrota. Is that any place for the first lesions of pellagra to appear? What a failure if they had been using women convicts for the experiment! But after all Dr. Lowery is right in advising the Goldberger diet, for there are many reasons.

Dr. Lowery calls pellagra a manifestation of an acidosis. Is it? Have you seen cases of acidosis? Yes—were they like pellagra? No. Everybody look up Acidosis. But still most pellagrins have "soured" on the world and the idea of too little acid in the stomach and too much and too many acids elsewhere may hang together. I will give a pellagrin an intravenous dose of Fischer's solution tomorrow and tell you the results next month.—EDITOR.]

